# The impact of shared sense of agency and moral dispositions on intergroup dishonesty

**DOI:** 10.1007/s12144-025-08026-0

**Published:** 2025-10-03

**Authors:** Giulio Piperno, Maria Serena Panasiti, Riccardo Villa, Salvatore Maria Aglioti

**Affiliations:** 1 Department of Psychology, Sapienza University of Rome and CLN2S@sapienza, Istituto Italiano di Tecnologia IIT, Rome, Italy; 2 IRCCS Fondazione Santa Lucia, Via Ardeatina 306, Rome, 00100 Italy; 3 Institute for Neural Information Processing, Center for Molecular Neurobiology (ZMNH), University Medical Center Hamburg-Eppendorf (UKE), Hamburg, Germany; 4 Department of Neurology, University Medical Center Hamburg-Eppendorf (UKE), Hamburg, Germany; 5 Department of Psychology, University of Rome “La Sapienza”, Via dei Marsi 78, Rome, 00185 Italy

**Keywords:** Morality, Honesty, Shared sense of agency, Deception, Intergroup interactions

## Abstract

Despite the societal impact of inter-group dishonesty, the specific circumstances and moral dispositions that influence this (immoral) behavior remain elusive. In this study, we examined the effect of different levels of shared Sense of Agency (s-SoA) with own team members and the type of moral dispositions on the tendency to deceive outgroups. To this end, we developed an online version of the Tone Tapping Task (TTT) to modulate s-SoA through partner predictability. Next, we run a within-subject experiment where 72 participants experienced a weak or strong s-SoA with their partner and then played the Multi-player Temptation to Lie Card Game, a moral task we devised to capture participants’ decisions to lie to increase their team’s monetary reward. Participants were presented with either favorable (*own team win*) or unfavorable (*other team win*) scenarios leading to low, medium, or high reward levels. Our analyses did not show a modulation of deceptive behavior based on the objective partner’s predictability. However, we found that lower s-SoA ratings with the partner and lower group-oriented “binding” morality were associated with an increased frequency of outgroup-favouring lies for lower rewards. Furthermore, participants with higher scores in dispositional “binding” morality showed a positive correlation between s-SoA levels and the likelihood of committing ingroup-favoring lies. These results contribute to existing frameworks by integrating Moral Foundations Theory with research on shared sense of agency, highlighting how subjective experiences of coordination can enhance the effect of group-centered moral tendencies.

**Supplementary Information:**

The online version contains supplementary material available at 10.1007/s12144-025-08026-0.

## Introduction

Dishonest behavior refers to the act of intentionally providing false or misleading information. Many factors affect the likelihood to deceive in individual settings, such as personal disposition (Hilbig, 2022; Panasiti et al., 2011), moral emotions (Parisi et al., 2021), or the magnitude of possible gain (Kajackaite & Gneezy, 2017; Scattolin et al., 2022). Conversely, the phenomenon of intergroup dishonesty, defined as the implementation of intentional deceptions aimed at benefiting one’s ingroup at the expense of an outgroup, has only recently captured the attention of cognitive scientists (Leib et al., 2021). While individual dishonesty is typically viewed as clearly immoral, determining what constitutes a moral action in intergroup contexts is not straightforward. This is because the benefits of lying may extend beyond the deceiver to other members of their group. As such, an individual choosing to deceive may weigh not only their own self-interest and the potential harm to the outgroup but also the potential benefits their actions could bring to ingroup members (Weisel & Shalvi, 2022). In line with this perspective, it has been shown that people deceive more when lying for their group compared to just for themselves (Gino et al., 2013; Halevy et al., 2015). Crucially, people seem to lie even when this does not increase their own payoff, but merely favors other ingroup members (Buckle et al., 2021; Cadsby et al., 2016), a phenomenon that can be modulated by social neurohormones like oxytocin (Shalvi et al., 2014). Parochial deception may be explained by psychological models that analyze morality from an evolutionary perspective (Atari et al., 2022; Janoff-Bulman & Carnes, 2013). Such theories claim that morality did not evolve as a device for universal collaboration, but rather as a cognitive system reinforcing cooperation among ingroup members and protecting against possible threats posed by other groups’ members (Conning, 2015). Consistently, the moral currency framework proposed by Weisel and Shalvi (Weisel & Shalvi, 2022) suggests that group dishonesty occurs when the moral value of collaboration with ingroup members overruns the moral norm of being honest. Nevertheless, groups may differ in inter-group dishonesty. For example, it was found that people are more willing to make dishonest decisions when loyalty among group members is strong, and competition with other groups is salient (Hildreth et al., 2016); when team members belong to the same social group (Cadsby et al., 2016); or when there is a risk of social exclusion (Thau et al., 2015). Moreover, individuals are more likely to engage in cheating and less likely to report others’ dishonest behavior when they are with a group that shares their social identity, compared to when they interact with outgroup members (Rullo et al., 2024).Thus, the type and the strength of bonds between members of the group appear to be crucial factors. In the current study, we tested whether experiencing different levels of shared Sense of Agency (s-SoA) with an ingroup member could modulate intergroup dishonesty, operationalized as the tendency to implement pro-ingroup lies in a moral decision-making task.

### Interactions of personal and shared sense of agency with moral behavior in group settings

The Sense of Agency (SoA) is defined as the experience of controlling one’s own actions and their consequences (Haggard, 2017). This feeling of control is thought to play a key role in the moral domain, as higher SoA is typically associated with greater personal and moral responsibility for harmful actions (Moretto et al., 2011; Spaccasassi et al., 2023). Indeed, it has been suggested that personal SoA (p-SoA) is crucial for experiencing a sense of responsibility over one’s actions and their outcomes (Frith 2013, 2014) and that assessing p-SoA levels could be fundamental in determining legal responsibility for immoral behavior (Haggard, 2017). While the majority of studies has focused on p-SoA, recent work has introduced the concept of a shared Sense of Agency (s-SoA), which arises when individuals coordinate actions to achieve a common goal (Pacherie, 2012). Recent theoretical and empirical work has highlighted that SoA is modulated in social contexts (Villa et al., 2022). Pacherie (2012) distinguished two forms of collective agency: **“we-agency**,**”** experienced when co-agents perform identical and synchronous actions (e.g., soldiers marching together), and **s-SoA**, which occurs when individuals perform complementary but distinct actions (e.g., two people moving a table together). In these scenarios, s-SoA adds to p-SoA without diminishing it, reflecting a shared control over the task. Building on these ideas, Silver and colleagues (2021) proposed that SoA in social contexts operates on a continuum, modulated by the level of cooperation and the nature of the relationship between co-agents. For instance, in hierarchical contexts, SoA may be perceived as “vicarious” for the leader and “violated” for the follower, leading to a reduction in p-SoA with significant implications in the moral domain. Consistently, Caspar and colleagues (2018) demonstrated that when participants are instructed to perform morally relevant actions in hierarchical settings, such as inflicting harm or financial loss, both leaders and followers experience a reduction in p-SoA. However, while hierarchical relationships in moral contexts tend to suppress p-SoA, the effects of egalitarian interactions—where no clear leader-follower roles exist—remain less understood. Importantly, previous studies did not investigate how s-SoA influences moral decision-making in non-hierarchical intergroup settings. According to the “continuum model” proposed by Silver and colleagues, in collaborative settings where s-SoA is expected to emerge, individuals may experience an increase in overall agency, extending it over their collaborative group. One first possibility is thus that experiencing high levels of s-SoA with ingroup members may enhance the sense of responsibility for decisions that affect the whole group. As a result, participants may reduce the frequency of immoral intergroup actions, such as harmful behavior toward outgroup members. This reduction in immoral behavior may be linked to a greater concern about the moral image of their group (Brambilla et al., 2013) or by higher levels of group-based guilt (Halevy et al., 2015). On the other hand, engaging in joint tasks can increase feelings of social closeness with co-agents (Tarr et al., 2018). Previous studies have shown that greater identification with one’s ingroup can lead to parochial behavior, where individuals prioritize the interests of their ingroup over the outgroup (Balliet et al., 2014; Swann & Buhrmester, 2015). Thus, a second possibility is that experiencing high s-SoA could enhance identity fusion with the partner, increase group identity salience, and ultimately lead to more pro-ingroup immoral behavior. In this context, decision-making within groups is often accompanied by diffusion of responsibility, which can reduce the emotional distress associated with potential negative outcomes of own actions (El Zein et al., 2019). This effect may arise due to mechanisms of moral disengagement (Bandura et al., 1996), having a loss of agency when actions are carried out collectively (Beyer et al., 2017), or through a shift in moral priorities, having the prioritization of ingroup gains becoming predominant in ethical considerations (Weisel & Shalvi, 2022).

Finally, moral behavior in general, and deceptive behavior specifically, can be strongly influenced by personal moral dispositions (Hilbig, 2022). The Moral Foundations Theory (MFT) (Graham et al., 2013) provides a useful framework to study morality in social context. According to the MFT, individuals possess several moral intuitions, or “Foundations.” The most recent model identifies six foundations: Care, Proportionality, Equality, Loyalty, Purity, and Authority (Atari et al., 2022). The first three foundations (Care, Proportionality, Equality) emphasize avoiding harm and promoting fairness, while the latter three (Loyalty, Purity, and Authority), grouped as the “binding moral foundations” (Atari et al., 2022; Graham et al., 2013), are more relevant to ingroup cohesion and intergroup conflict (Hadarics & Kende, 2018; Smith et al., 2014). The personal disposition can modulate intergroup moral interactions (Reed & Aquino, 2003). Thus, a third possibility is that individuals with a less group-oriented (binding) moral disposition would align more with the first hypothesized process, behaving more prosocially due to a greater sense of responsibility for their own group. In contrast, individuals with a more group-oriented moral disposition would likely exhibit a behavior aligned with the second hypothesized process, giving relevance to the group identity and prioritizing ingroup interests. To understand the interactions between s-SoA, personal moral disposition, and intergroup dishonesty, we conducted two online studies.

### The current research

We conducted two studies: a preliminary study to validate a task designed to induce changes in shared Sense of Agency (s-SoA) in an online setting, and a main study to examine how variations in s-SoA influence moral behavior in group contexts. In the preliminary study, our goal was to validate an online paradigm capable of eliciting different levels of s-SoA in participants, ensuring its effectiveness for use in the main experiment. Bolt and Loehr (Bolt et al., 2016; Bolt & Loehr, 2017) demonstrated that the predictability of the co-agent in a coordination task can modulate the s-SoA. To this aim they developed the Tone Tapping Task (TTT), a paradigm where participants collaborate to produce a sequence of tones maintaining a rhythm initially provided by a metronome. Each participant is paired with a simulated partner whose timing is manipulated to be either predictable—consistently matching the metronome’s pace—or unpredictable—varying by accelerating or decelerating between tones. The authors demonstrated that participants experience a stronger s-SoA over actions and outcomes, when interacting with the predictable partner compared to the unpredictable one. However, when measuring SoA, they used a unidimensional scale ranging from p-SoA to s-SoA. Thus, it was not possible to disentangle how much the effect was due to an increase of s-SoA or to a decrease of p-SoA. In this preliminary study we tested the validity of an online adaptation of Bolt and Loehr’s (Bolt et al., 2016; Bolt & Loehr, 2017) paradigm while measuring s-SoA and p-SoA separately, at the end of each block with two aims: (i) replicate previous findings of high (*low*) s-SoA when the coordinating partner is predictable (*unpredictable*) (Bolt & Loehr, 2017); (ii) confirm that the effect is driven by changes of s-SoA rather than p-SoA, which in turn should not be affected. The aim of the main study was to analyse the tendency to deceive for a monetary reward in an intergroup setting, after the induction of either low or high s-SoA with the partner of the TTT, acting as a team member. To measure the tendency to lie, we created a novel Multiplayer Temptation to Lie Card Game (MP-TLCG), in which we adapted for a multi-player scenario a paradigm originally developed for testing deception for personal gain (Azevedo et al., 2018; Panasiti et al., 2011, 2014, 2016; Parisi et al., 2021; Scattolin et al., 2022; Schepisi et al., 2020). This task is designed to elicit a moral conflict between the opportunity to deceive to increase one’s group monetary payoff and behave honestly. Finally, to explore the possible modulatory effects of moral disposition, we asked participants to fill out the Moral Foundation Questionnaires 2 - MFQ-2, (Atari et al., 2022) before the experimental session.

## Preliminary study

The aim of the preliminary study was to validate the Tone Tapping Task (TTT) in an online setting. We predicted that participants in the predictable partner condition would experience higher shared Sense of Agency (s-SoA) compared to those in the unpredictable partner condition, while personal Sense of Agency (p-SoA) would remain unaffected. We also aimed to examine whether this effect varied across blocks or remained stable over time.

## Methods

### Participants

The sample consisted of 60 participants (49 females, mean age ± SD: 21 ± 3), all of whom were over 18 years old and thus considered capable of providing informed consent independently according to Italian law. They were recruited among the students of the Faculty of Medicine and Psychology of the University of Rome “La Sapienza”. The sample size was determined based on previous studies using the original version of the adopted paradigm (Bolt & Loehr 2016, 2017). All the participants received exam credits as compensation for their participation. Participants were naïve with respect to the purposes of the study. The requirements to participate in the study were being an Italian speaker, having a stable internet connection, and not being affected by neurological or psychiatric conditions.

### Procedure

The experimental protocol was approved by the Ethics Committee of “La Sapienza University of Rome” and was carried out following the principles of the Declaration of Helsinki. Due to the Covid-19 restrictions occurring at the time of the testing, between April and June 2021, the entire procedure was carried out online. After reading and providing informed consent, participants were given a specific appointment to participate in the experiment via email. We told participants that time slots were necessary to allow other participants to connect to our platform, simultaneously. They received an email with the link to the experiment right before the agreed time. Before starting the tone-tapping task (see below), participants were assigned to a coloured avatar icon, while their partner was assigned another one. After completing the first session, participants were asked to notify the experimenter. After 10 minutes, they received the link to perform the second session. The online platform Testable was used to run the experiment (Rezlescu et al., 2020).

### Tone tapping task

**Fig. 1:**
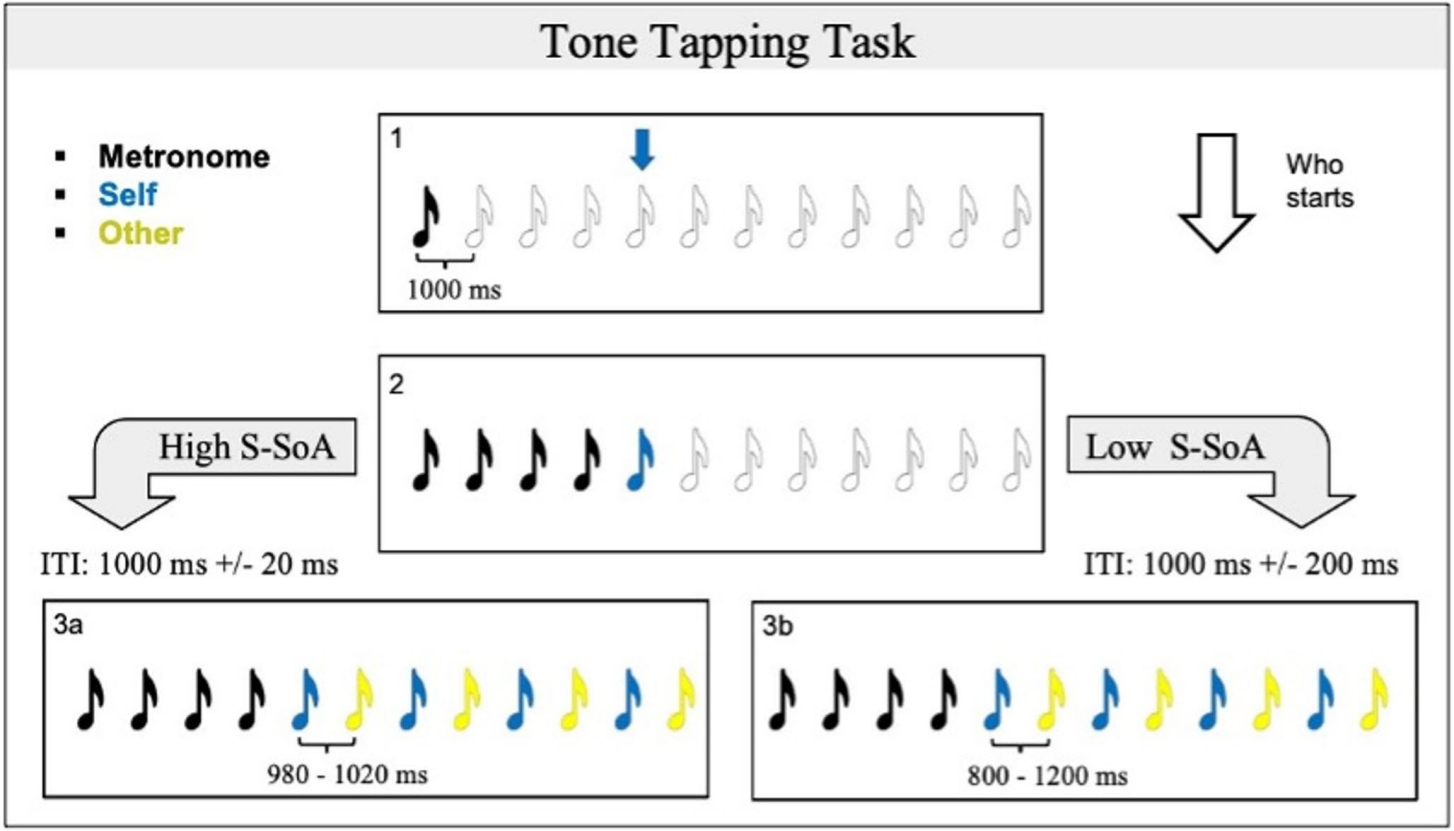
Schematic representation of the TTT. (1) The first 4 tones are played by the metronome, with a fixed 1000 s interval between each tone. Simultaneously with each tone, the notes get colored with black. An arrow over the 5^th^ tone indicates the player who has to start the sequence. (2) The tones from 5^th^ to 12^th^ are played by the two players (the participant and the simulators). The notes change color to match the current player’s avatar color, depending on the turn. (3a) In the High s-SoA condition, the simulator behavior is predictable, with the inter-tone- interval varying between 980 and 1020 ms. (3b) In the Low s-SoA condition, the simulator behavior is unpredictable, with the inter-tone- interval varying between 800 and 1200* ms*

The tone tapping task (TTT) is an online adaptation of the paradigm developed by Bolt and colleagues (Bolt et al., 2016; Bolt & Loehr, 2017). This paradigm was developed following theoretical suggestions (Pacherie, 2012), that joint actions most likely boost s-SoA, if they are executed by (i) co-agents having little role specialization such that co-agents perform similar actions, (ii) by a group consisting of a fixed and small number of (ideally two) co-agents, and (iii) where a hierarchy is absent. In the TTT, the participant had to coordinate with a partner (in reality, a computer simulating another participant’s behavior, see below) to produce an alternating sequence of tones, maintaining a specific rhythm of 1 s interval between the tones (Fig. 1). Each tone had a duration of 100 ms. At the beginning of each trial, after the presentation of a fixation cross lasting 2000 ms, 12 uncoloured images, representing notes, were displayed horizontally. A metronome played the first four tones. The notes, starting from left to right, turned black each time the corresponding tone was produced. The appearance of blue or yellow arrow over the fifth note, indicated which player had to play the subsequent tone for the current trial. After that, the two players had to continuously alternate with each other in generating the tones, until the last note. Participants had to press the spacebar to produce the tone and the colour change of the note. The predictability of the partner was manipulated within subjects across the two experimental sessions, as each participant was paired with a predictable and with an unpredictable partner. To manipulate the partner’s predictability, we adjusted the timing of their responses to participants’ actions. Thus, the latency between the participants’ tone and the simulator tone, the inter-tone intervals (ITIs), varied across the two conditions. These ranged from 980 to 1020 ms (i.e., the 1000 ms metronome pace ± 20 ms) in the predictable condition, and from 800 to 1200 ms in the unpredictable one (1000 ms metronome pace ± 200 ms). Participants completed two separate sessions: one with a predictable partner and the other with an unpredictable partner. Each session was divided into five identical blocks, and within each block, participants performed six consecutive trials. After completing each block, participants had to rate the level of control they experienced over the sequence, indicating the personal agency rating - p-SoA and shared agency ratings- s-SoA with the partner. Participants expressed their ratings by selecting a value ranging from 0 to 100 on separate Visual Analogue Scales (VAS). We also asked the rating of the partner ‘s predictability as manipulation check. To measure the impact of partner predictability over the social perception of the partner across the two conditions, at the end of both sessions, participants rated their feeling of Social Closeness with the other player, and their inclusion of the other in the self with an adapted IOS (Aron et al., 1992). To measure an index of Social Closeness, similarly to Tarr and colleagues (Tarr et al., 2018), we asked three questions to address Sympathy, Attunement, and Similarity using VAS. All the questions are summarized in Table 1. Finally, since the current emotional state can influence the prevalence of dishonest behavior (Parisi et al., 2021), we also measured the positive and negative affect (Diener & Emmons, 1984) at the end of each session, to ensure the absence of potential difference in the two conditions. The order of the two partners’ predictability sessions was counterbalanced across participants.

**Table 1:** This table reports the VAS scales participants were requested to fill: the p-SoA and s-SoA items at the end of each block and the Sympathy, Attunement, Similarityitems at the end of each session.

Measure	Questions
From 0 (completely not) to 100 (completely) how much did you feel…	
p-SoA	… to control the sequence of produced sounds?
s-SoA	… to share the control of the produced sequence of sounds with the other player?
Sympathy	… sympathy for the other player.
Attunement	… in tune with the other player?
Similarity	… similar to the other player?
Manipulation Check	… that you could predict when the other player would generate a sound

### Data analysis

Data analysis was performed with R. For the manipulation check and SoA measures, we ran separate repeated measures ANOVAs. We treated each participant’s Block as a separate observation, obtaining ten observations per participant (5 per condition). We used s-SoA and p-SoA (self- and shared-agency) ratings as continuous dependent variables. Each model included Condition (Predictable; Unpredictable) and Block number as categorical predictors, as well as their interaction. We inserted block number and its interaction with Condition to identify the number of blocks necessary to obtain a consistent modulation of s-SoA. Post hoc comparisons were performed applying False Discovery Rate (FDR) correction. To obtain a composite value of Social Closeness, for each participant we computed the mean of the scores given to the questions on sympathy, attunement, and similarity. Since IOS, Social Closeness and PA and NA measures were taken just once per session, we used two-tailed paired t-tests with Condition as the independent variable. When significant, effect sizes were calculated using Cohen’s d.

## Results

As expected, Participants rated the predictable partner as significantly more predictable than the unpredictable one, confirming the perceived difference between the two Conditions (F (1,59) = 49.94, *p* < 0.001, η^2^ = 0.123). Concerning s-SoA, both Condition (F (1,59) = 22.11, *p* < 0.001, η^2^ = 0.064) and Block number (F (4,236) = 7.60, *p* < 0.001, η^2^ = 0.010) were significant predictors. Participants gave a higher rating of s-SoA when they coordinated with the high-predictability (EMM: 72.8, SE: 2.85) compared to the low-predictability partner (EMM: 60.2, SE: 2.90; d = 0.52, b = 12.60, t = 4.70, *p* < 0.001). In addition, in both conditions, we found an increase in the rating of s-SoA associated with the increase of the block number, which was statistically significant when comparing the first and the last block (post hoc between first and last block: d = 0.30, b = 7.40, t = 4.79, *p* = 0.001). The interaction between Condition and Block was not significant (F (4,236) = 0.39, *p* = 0.81). Concerning p-SoA, there was no significant difference between Conditions (F (1,59) = 1.93, *p* = 0.17) indicating that there was no change in p-SoA due to the predictability of the other player. Also in this case, we found a significant effect of Block (F (4,236) = 4.65 *p* = 0.004, η^2^ = 0.005), characterized by a similar stepwise increase of p-SoA (post hoc between first and last block: d = 0.2, b = 4.44, t = 2.85, *p* = 0.02). The results of the paired t-tests concerning IOS, and Social Closeness were both significant (IOS: d = 0.44, b = 0.68, t = 4.7591, *p* < 0.001, Social Closeness: d = 0.35, b = 8.23, t = 3.47, *p* < 0.001), showing that participants displayed more Social Closeness and other-in-the-self inclusion with the predictable vs. the unpredictable partner. Finally, both Positive and Negative affect were non significantly different across the two conditions (PA: t = 0.24, *p* = 0.8, NA: t = 0.11, *p* = 0.91). Please refer to Supplementary Information, Figure S1 for a graphical representation of the results reported in this section.

## Preliminary study discussion

In this preliminary study, we aimed to develop an online adaptation of the tone tapping task (Bolt & Loehr, 2017), the finding that s-SoA is modulated by the predictability of a partner’s actions. The results were in line with previous findings (Bolt & Loehr, 2017), with stronger s-SoA when interacting with a predictable vs. unpredictable partner. In addition, we found that both p-SoA and s-SoA increased in consecutive blocks, irrespective of partner’s predictability. This could be interpreted as a general improvement in SoA following practice. Indeed, a similar improvement in SoA following training has been documented in previous studies (van der Wel et al., 2012). Moreover, the interaction between predictability condition and block number resulted not significant. This suggests that our manipulation was effective already in the first block and that the difference in s-SoA ratings between the two conditions remained stable over time. Importantly, by measuring p-SoA and s-SoA separately, we verified that the manipulation of partner predictability specifically affected the latter. In line with our hypothesis, we also found that interacting with a more predictable partner was associated with more social closeness and inclusion of the other in the self. Having established that the TTT successfully manipulated s-SoA (as well as social closeness and IOS) depending on the partner’s predictability, we performed a second study, where we examined whether experiencing higher or lower s-SoA levels may affect the tendency to deceive in the following intergroup moral task.

## Main study

The results of the preliminary study validated the Tone Topping Task (TTT) as an effective tool for manipulating s-SoA in an online environment. In the main study, we aimed to explore whether this modulation of s-SoA was associated with moral behavior in an intergroup context. After completing the TTT, participants engaged in the Multiplayer Temptation to Lie Card Game (MP-TLCG), where their previous partner from the TTT was designated as their ingroup member. In the MP-TLCG, participants had the option to either lie or tell the truth, with opportunities to increase their group gain at the expense of an outgroup. Each trial was associated with varying reward levels (low, medium, or high). We examined whether moral behavior was influenced by both the objective predictability of the partner and participants’ subjective experience of s-SoA with the partner. Additionally, we considered whether moral disposition played a modulatory role in these behaviors.

## Methods

### Participants

To determine the sample size, we ran a power analysis on a pilot study of 52 subjects, using *mixed-power* simulation method (Kumle et al., 2021). Considering the three-way interaction of interest, a sample size of seventy-two participants was suggested to achieve a power of 0.8. We tested seventy-six participants four of which were discarded for technical reasons (connection failure during the experiment). The final sample consisted of seventy-two participants (self-referred as female 35, as male 35, as other 2), mean age ± SD: 29 ± 9 years old. For the analysis of the interaction between moral disposition and the experimental manipulation only, we had to exclude two participants due to a technical issue that prevented their MFQ-2 responses from being recorded. All subjects were paid a minimum of 12 € for their participation in the study. Depending on their decisions in the Multiplayer-Temptation to Lie Card Game (MP-TLCG, see below), participants’ team had the possibility of receiving a bonus up to 2 € per session which would have been split in half with their partner in the current session - thus participants could receive up to 1 € of bonus per session. Participants were recruited through Prolific, an online platform designed to perform online experiments (Palan & Schitter, 2018). The requirements to participate were being an Italian speaker, not having taken part in the preliminary study and previous experiments involving the use of the TLCG, and not being affected by neurological or psychiatric conditions.

### Procedure

The experimental procedure was conducted online. Participants were first requested to read and approve the informed consent and, immediately after, to fill out the Moral Foundation Questionnaire 2 (MFQ-2). Participants were then contacted via Prolific and asked to suggest an appointment for the experimental session connection within a week. At the agreed day and time, participants opened the corresponding link on Prolific and they were assigned an avatar. Then, they were entered into an online waiting room where they saw 3 other participants (in reality, computer-controlled) progressively entered the waiting room, each of them being assigned an avatar of a different colour. The experiment started after the number of connected participants reached 4. Participants performed the TTT, which aimed to generate a high s-SoA with one partner and a low s-SoA with the other. The last session of the TTT with each of the two partners was followed by a session of the MP-TLCG, which allowed to measure the tendency to lie to another group of participants, while having the partner of the TTT as their team member. At the end of the experiment, participants answered a series of control questions aimed at measuring how much they perceived they were interacting with other real persons during the tasks and compiled the MFQ-2. The online platform Testable was used to run the experiment (Rezlescu et al., 2020). The experimental protocol was approved by the Ethics Committee of La Sapienza University of Rome and was carried out in accordance with the principles of the Declaration of Helsinki.

## Materials

### Moral Foundation Questionnaire-2 (MFQ-2)

The MFQ-2 is a 36-item questionnaire measured on a 5-point Likert scale, subdivided in six sub-scales It is designed to assess the extent to which individuals rely on the six different moral categories when making judgments about morally relevant situations (Atari et al., 2022). The MFQ-2 allows to gauge the following six domains of the Moral Foundation Theory (revised): Harm/Care (concerns regarding care and protecting individuals from harm), Fairness/Proportionality (the psychological motive for rewards and punishments to be proportionate to merit and deservingness), Equality (the psychological motive for balanced reciprocity, equal treatment, and equal outcome), Ingroup/Loyalty (the tendency to prioritize group identity, loyalty, and patriotism), Authority/Respect (the desire and respect for social hierarchies, authority, and the maintenance of order), Purity/Sanctity (the psychological motive for the preservation of purity, cleanliness, and sacredness). To obtain a single value for Binding Morality (BM), we calculated the average of the scores given to the subscales of Ingroup/Loyalty, Authority/Respect and Purity/Sanctity, as done in previous studies on an Italian sample (Di Battista et al., 2018).

### Tone tapping task

**Fig. 2:**
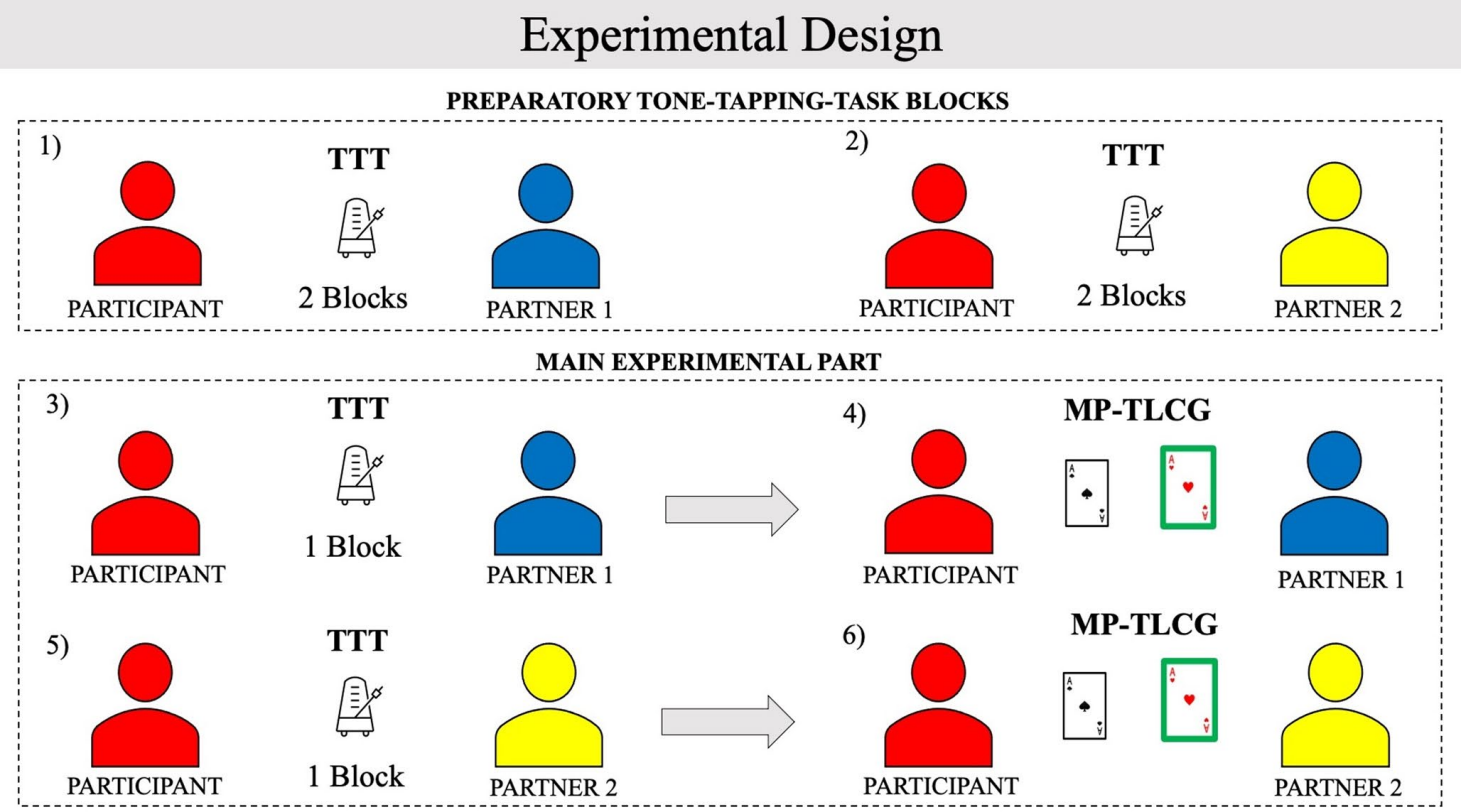
Sequence of the tasks executed by the participants in the main study. They first performed 2 blocks of the Tone Tapping Task (TTT) with the first partner (1) and two blocks of the TTT with the second partner (2), as preparatory phase to familiarize with both players. Then they performed the last block of the TTT with the first partner (3), immediately followed by the Multi-Player Temptation to Lie Card Game (MP-TLCG) with the first partner. Finally, they performed the last block of the TTT with the second partner (5), followed by the MP-TLCG with the second partner (6)

As in the preliminary study, we asked participants to perform the TTT to elicit different levels of s-SoA with a predictable or unpredictable partner. We made minor modifications to the paradigm in the second study to streamline the procedure and enhance the contrast in s-SoA ratings between high and low predictability conditions. Since the addition of the MP-TLCG significantly increased the experiment’s duration and the preliminary study showed that s-SoA differences were stable across blocks, we reduced the number of blocks per condition from five to three. Additionally, to emphasize the distinction between the two partners and ensure participants experienced both predictability conditions before the MP-TLCG, we alternated high- and low-predictability blocks rather than presenting them sequentially, as done in the preliminary study. Specifically, participants first performed two blocks with the predictable (*unpredictable*) partner (Fig. 2.1), then two blocks with the unpredictable (*predictable*) partner (Fig. 2.2). The order of the two conditions (high, low predictability) was counterbalanced across participants. Afterwards, participants performed one additional TTT block with each partner (Fig. 2.3 and Fig. 2.5) before starting each session of the MP-TLCG (Fig. 2.4 and 3.6). Since both the predictable and unpredictable partners separately acted as team members in MP-TLCG sessions, these additional TTT sessions served as “reminder” blocks, helping to maintain higher or lower levels of s-SoA, depending on the partner’s predictability. To favor the discrimination between the high and low predictability partners, the two avatars had different colours (blue or yellow). The measures of SoA (p-SoA, s-SoA ratings) were collected at the end of each block by means of VAS, while the measures of Social Closeness, IOS, and positive and negative affect were collected at the end of the second block, and after the reminder blocks, for each predictability condition (for a graphical description of the procedure see Fig. 2).

### Multi-player temptation to lie card game (MP-TLCG)

**Fig. 3:**
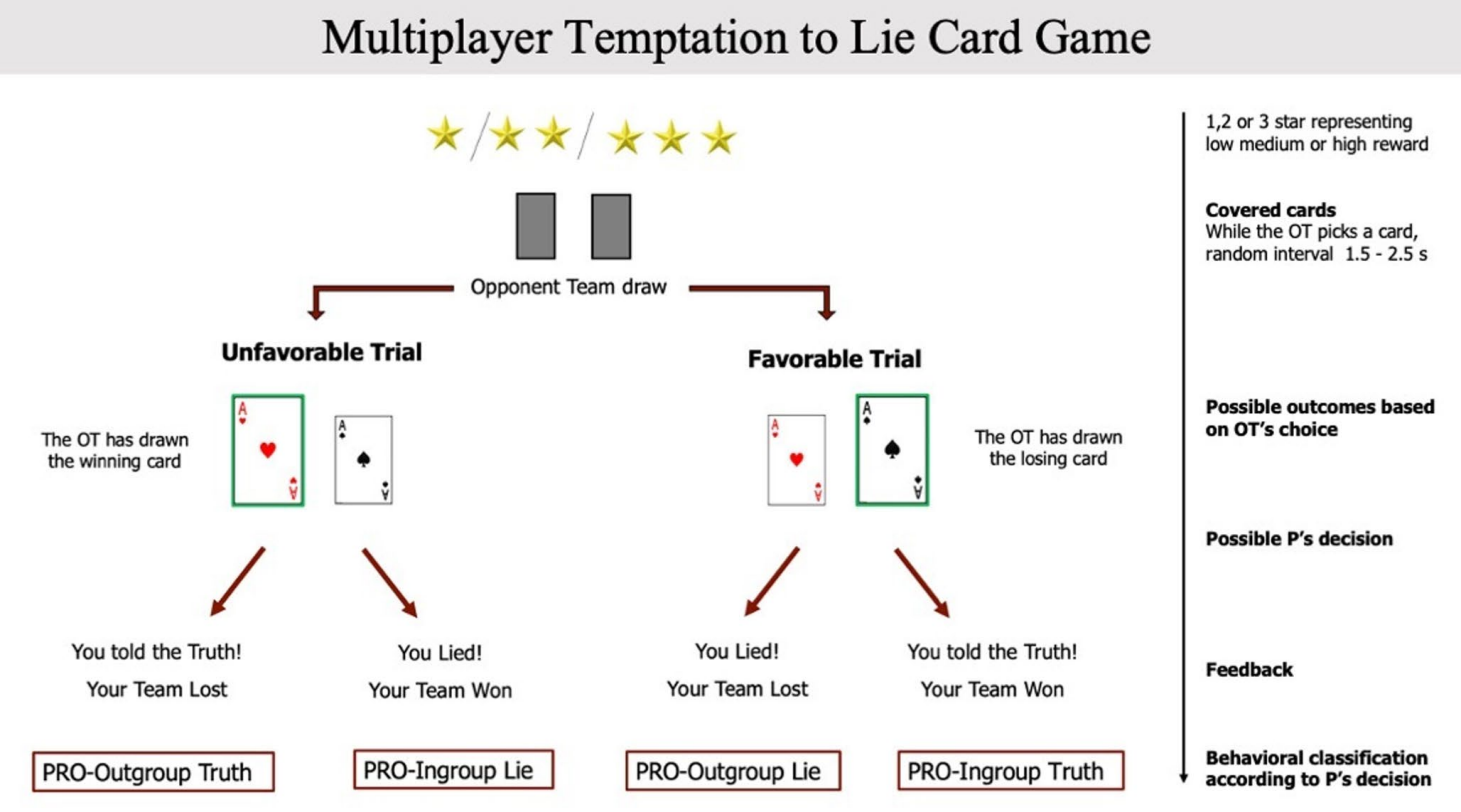
Schematic representation of the MP-TLCG.The Opponent Team (OT) choose the cards with two possible outcomes (Favorable or Unfavorable for the participant). The Participant (P) can tell the truth or lie. Then feedback is provided for each response, for all the four possible scenarios. The number of stars represent the reward obtainable for each trial. The timeline of each trial is provided in the rightmost part of the figure

The MP-TLCG is an adaptation of the TLCG (Azevedo et al., 2018; Panasiti et al., 2011, 2014, 2016; Parisi et al., 2021; Scattolin et al., 2022; Schepisi et al., 2020; Vabba et al., 2022), a task designed to elicit in participants a moral conflict between the opportunity to deceive and increase one’s monetary payoff or behave honestly (Fig. 3). While the original version of the TLCG involves two individuals, the MP-TLCG extends the examination of spontaneous deception to inter-group contexts, by allowing the presence of two teams competing for a monetary win. The gain of one team leads to the loss of the other, and the resulting reward is always shared with the team partner. While this arrangement complicates the distinction between immoral acts committed for personal versus group gain, such design more accurately reflects real-life interactions, where immoral actions directed at out-groups typically result in benefits for all ingroup members. Here, one team was composed of the participant and their partner from the previous session of the TTT (Participants’ Team - PT). The participant always had the role of the “Decider”, while their partner had the role of the “Observer”. The opponent team (OT) was formed by two anonymous other players, both having the roles of “Card Drawers”. The task consisted of a two-cards game, where an ace of hearts was associated with monetary gain, and an ace of spades with monetary loss. We told participants that the monetary gain was always split between the two members of the winning team, thus all the reward accumulated during the task was going to be split with the team partner. We manipulated the reward levels, since previous literature partially supports the presence of a significant relationship between dishonesty and obtainable payoff (Gerlach et al., 2019; Scattolin et al., 2022). Thus, the possible gain in each trial was variable. It could be either small, medium, or high, as represented on screen by one, two, or three stars. The use of different reward levels was also included to promote participants involvement in the task and to help the prevention of automatic response patterns. Moreover, the varying reward levels could favor the emergence of more strategic behaviors, potentially leading participants to act more flexibly– as in real life. For example, participants might act more pro-ingroup when higher rewards were at stake and more pro-outgroup when lower rewards were involved, thereby balancing the number of wins and losses while maximizing their group’s economic gain. Participants were not aware of the precise magnitude associated with each reward level to avoid the use of counting based cumulative strategies. Participants were told that, for each trial, a member of the OT would pick one of two covered cards, without knowing the outcome of the choice. If the OT picked the ace of hearts (*spades*), this indicated that the OT had won (*lost*) and that the PT had lost (*won*) in that trial. Hence, the outcome of a trial could be “favorable” or “unfavorable” for the PT, depending on whether the OT picked the ace of spades or hearts, respectively. Each trial began with the observation of the two covered cards. Then, after a random interval between 1500 and 2500 ms, the card picked by the OT was revealed, by showing either ace of spades or hearts as increased in size. The left/right position of the ace of hearts/spades was counterbalanced across trials. The participant, who had the role of “Decider”, could confirm the outcome of the trial, by telling the truth, or reversing it, by telling a lie. When the participant decided to confirm the outcome– i.e., honest behavior– this could either lead their team to win when they should have (pro-ingroup truth) or to lose when they should have lost (pro-outgroup truth). When the participant decided to reverse the outcome– i.e., dishonest behavior– they could lead their team to win when they should have lost (pro-ingroup lie), or to lose when they should have won (pro-outgroup lie). To make their choice, participants had to press either the button ‘‘A’’ or ‘‘L’’ of a keyboard, which allowed them to communicate to the OT that they had picked the winning or losing card. After each decision, a feedback message appeared on the screen communicating to the PT the outcome of the trial. Depending on the opponents’ pick (Favorable or Unfavorable for the PT) and on participant’s decision (to tell a lie or the truth), there were four possible messages: “You lost, you told the truth!”, “You won, you told the truth!”, “You lost, you lied!”, “You won, you lied!”. We told participants that the other member of their team, having the role of the “Observer”, could see the card picked by the OT, and their decisions in each trial, without interfering. We also told participants that the OT had drawn the cards in a previous experimental session, and their answers had been pre-recorded. Participants were told that players from the opposing group would not be informed about decisions made during each trial of the game, but only about the final economic gain of their team. They were told instead that their partner would be observing single-trial decisions. As for the TTT, in reality there were no other participants, and a computer simulated the OT picking cards. The experimental design comprised 6 conditions, resulting from the combination of factors Outcome (Favorable, Unfavorable) and Reward level (low, medium, high). Each condition was repeated in 16 trials, for a total of 96 trials per MP-TLGC session. The MP-TLCG was repeated twice, where the participant team player was either the predictable or unpredictable partner of the TTT.

### Final debriefings

At the end of each MP-TLCG session, we asked participants to answer a series of VAS (0 to 100) concerning the SoA they experienced in the MP-TLCG, their feeling of personal and shared responsibility over their choices - how responsible they felt for the decisions made in the game, and to what extent they believed responsibility of own team’s decisions was shared with their partner - the impact of the presence of their partner on their decisions, and their sense of Social Closeness with the latter. At the end of the experiment, we asked the participants how much (from 0 to 100) they had the impression to have played with real other participants during the task, separately for both predictable and unpredictable partner.

## Data analyses

### Tone tapping task

To evaluate the effectiveness of the manipulation, data analysis for SoA ratings was performed as in the preliminary study.

### Multiplayer temptation to lie card game

Data analysis was performed with R. We ran binomial multilevel mixed log-linear regression analysis (LMM or ‘mixed effects models’, (Garson, 2013; Bates et al., 2014). We treated each participant’s decision as a separate observation, obtaining 192 observations per participant (96 per MP-TLCG session). We had one categorical dependent variable (Lie/Truth) and a different set of predictors depending on whether the analysis focused on the objective level of partner’s predictability (low, high) or the subjective s-SoA levels. Indeed, an inspection of the s-SoA data revealed that 15 subjects, consisting of 20% of our sample, displayed a null or reversed pattern with respect to other participants, with higher (or equal) levels of s-SoA in high and low predictability conditions. In light of the variability in s-SoA levels within the two conditions, we decided to build two separate statistical models, which either comprised Condition or s-SoA ratings given for the block preceding the MP-TLCG as the agency related variables. Therefore, one model had Outcome (Favorable; Unfavorable), Reward (Low; Medium, High), and Condition (Predictable; Unpredictable) as categorical predictors and Binding (scaled) as the continuous predictor; the other had Outcome (Favorable; Unfavorable) and Reward (Low; Medium, High) as categorical predictors and Binding and s-SoA (both scaled) as a continuous predictor. It is worth noting that Leib and colleagues (Leib et al., 2021) demonstrated that in group dishonesty studies, the likelihood of deception is influenced by the presence of experimental deception, i.e., participants may be suspicious concerning the real existence of other players, and in turn this could affect their decision. To control for the possible influence on decisions to lie due to the belief that the other players were actually present, in each model we added participants answers to the corresponding control question as covariate (Believe Manipulation). In each model, participants were entered as grouping factors (i.e., random intercepts), with fixed effects and their interactions modelled as random slopes over participants (Barr et al., 2013). For each model, we iteratively excluded the random part explaining the least variance, until reaching the most complex converging models (Bates et al., 2015).

Following this procedure, the resulting converging model when including Condition as categorical predictor was as follows:

$$\begin{array}{c}Lies\sim\left(Outcome\ast Condition\mid Subject\right)+Believe\;Manipulation+\\Outcome\ast Condition\ast\;Reward\ast Binding\;Morality\left(BM\right)\end{array}$$

When including s-SoA, we employed the following, converging model:

$$\begin{array}{c}Lies\sim\left(Outcome\ast s-SoA\mid Subject\right)+Believe\;Manipulation+\\Outcome\ast s-SoA\ast\;Reward\ast Binding\;Morality\left(BM\right)\end{array}$$

We tested these models with the type-III anova. When an interaction resulted significant, post-hoc pairwise comparisons using estimated marginal means were performed applying FDR correction for multiple comparisons.

## Results

### Tone tapping task– manipulation check

Concerning s-SoA, as expected, the main effect of Condition (F (1,71) = 50.36 *p* < 0.001) was significant, with an overall higher level of s-SoA when playing with the predictable vs. unpredictable partner (b = 18.80, t = 7.10, *p* < 0.001). Thus, manipulating partner predictability was overall effective in modulating the experience of s-SoA. Still, around 20% of the sample (*n* = 15) revealed null or reverse pattern, suggesting the presence of a certain variability among participants. The interaction between Condition and Block was also significant (F (2,142) = 6.30, *p* = 0.014, η^2^ = 0.016). Post-hoc comparisons revealed that while there was no change of s-SoA in the predictable condition, there was a significant decrease between the second and the third block in the low-predictability condition (b = − 5.88, t = − 2.31, *p* = 0.022). This led to an increase in the effect size of Condition from the second block (d = 0.65, b = 14.80, t = 5.32, *p* < 0.001) and the third block (d = 0.89, b = 22.70, t = 6.78, *p* < 0.001). A similar pattern emerged with measures of IOS and Social Closeness. As expected, IOS and Social Closeness were significantly different among Conditions (IOS: F (1,71) = 76.92, *p* < 0.001, Social Closeness: F (1,71 = 65.12, *p* < 0.001)), with higher ratings in the predictable condition (IOS: b = 1.29 t = 8.77, *p* < 0.001, Social Closeness: b = 17.50, t = 8.07, *p* < 0.001). We found a significant interaction between Condition and Block (IOS: F (1,72) = 13.85, *p* < 0.001, η^2^ = 0.033, Social Closeness: F (1,72) = 8.85, *p* = 0.001, η^2^ = 0.014). Post-hoc comparison revealed an increase in the difference between predictable and unpredictable conditions between the second block (IOS: d = 0.57, b = 0.97, t = 6.10, *p* < 0.001, Social Closeness: F (d = 0.67, b = 14.60, t = 6.12, *p* < 0.001) and the block preceding the MP-TLCG (IOS: d = 1.04, b = 1.61, t = 8.91, *p* < 0.001, Social Closeness: F (d = 0.90, b = 20.50, t = 8.54, *p* < 0.001). Again, in both cases the interaction was driven by a decrease in values in the final block of low predictability condition (IOS: b = − 0.51, t = 3.57, *p* < 0.006, Social Closeness: b = − 7.13, t = 3.57, *p* < 0.006). Contrary to the previous study, this time we found a significant difference in p-SoA between the two conditions, due to lower values when interacting with the unpredictable partner (d = 0.26, b = 5.53, t = 3.7, *p* = 0.003). Finally, Positive affect was marginally significant (t = 1.90, *p* = 0.051) with a negligible effect size (d = 0.18) while Negative affect was not significantly different across the two conditions (t = 1.70, *p* = 0.08).

Please refer to Supplementary Information, Figure S2 for a graphical representation of the results reported in this section.

### Multiplayer temptation to lie card game

#### Objective manipulation of partner predictability

As expected, there was a significant main effect of Outcome (χ2 = 35, *p* < 0.001), such that participants lied more in the unfavorable scenario (estimate = 7.23, SE = 0.8, z = 8.96, *p* < 0.001). This effect was modulated by a significant interaction with reward level (χ2 = 159.96, *p* < 0.001), with a stronger tendency to lie for higher rewards in unfavorable scenarios (R1 - R2: estimate = − 1.45, SE = 0.10, z = −13.41, *p* < 0.001; R2-R3: estimate = −0.75, SE = 0.10, z = −7.50, *p* < 0.001) and lower rewards in favorable scenarios (R1 - R2: estimate = 0.83, SE = 0.14, z = 5.85, *p* < 0.001; R1-R3: estimate = 0.86, SE = 0.14, z = 6.02, *p* < 0.001). Contrary to our predictions, we found no significant main effect of Condition, nor interactions involving the Condition factor (all *p* > 0.1).

#### Subjective agency ratings

As in the previous analysis, the main effect of Outcome (χ2 = 25.17, *p* < 0.001) and the interaction between Outcome and Reward (χ2 = 270.94, *p* < 0.001) were significant. In addition, we found significant triple interactions between s-SoA, Outcome and Reward (χ2 = 11.26, *p* = 0.004) (See Fig. 4.1), between BM, Outcome, and Reward (χ2 = 8.50, *p* = 0.014) (See Fig. 4.2) and between s-SoA, BM and Outcome (χ2 = 4.30, *p* = 0.038) (See Fig. 4.1). The post-hoc comparisons revealed that for both interactions involving reward, thus s-SoA * Outcome * Reward and BM * Outcome * Reward, the effect was mainly driven by pro-outgroup lies. Indeed, for low vs. medium and high rewards, either at weaker levels of s-SoA or BM, participants were more likely to reverse favorable outcomes for their team– i.e., increased pro-outgroup lies (S-SoA: R1 - R2 estimate = −0.69, SE = 0.15, z = −4.50, *p* < 0.001; R1-R3 estimate = −0.60, SE = 0.15, z = −4.00, *p* = 0.001, BM: R1 - R2 estimate = −0.43, SE = 0.17, z = −2.54, *p* = 0.016; R1-R3 estimate = −0.75, SE = 0.17, z = −4.30, *p* < 0.001). The slope for low reward (R1) was significantly different from 0 for BM (estimate= −1.09, SE = 0.49, z = −2.22, *p* = 0.026) but not for s- SoA (estimate= −0.77, SE = 0.51, z = −1.48, *p* = 0.13). There was no significant effect concerning pro-ingroup lies (all *p* > 0.2). Concerning the interaction s-SoA * BM * Outcome, the post-hoc comparison revealed that the increase in dispositional ingroup Binding morality was associated with the increase of the positive relationship between s-SoA and the likelihood to lie in the unfavorable condition (z = 2.50, *p* = 0.012). Such positive relationship was significant only for people with high dispositional BM (+ 1 SD), for whom the increase of s-SoA came with a higher likelihood of telling pro-ingroup lies (estimate = 1.42, SE = 0.65, z = 2.40, *p* = 0.016). Supplementary analyses on BM subdimensions revealed that this interaction was mainly driven by Purity and Loyalty, while Authority showed no significant contribution (see Supplementary S5).

**Fig. 4:**
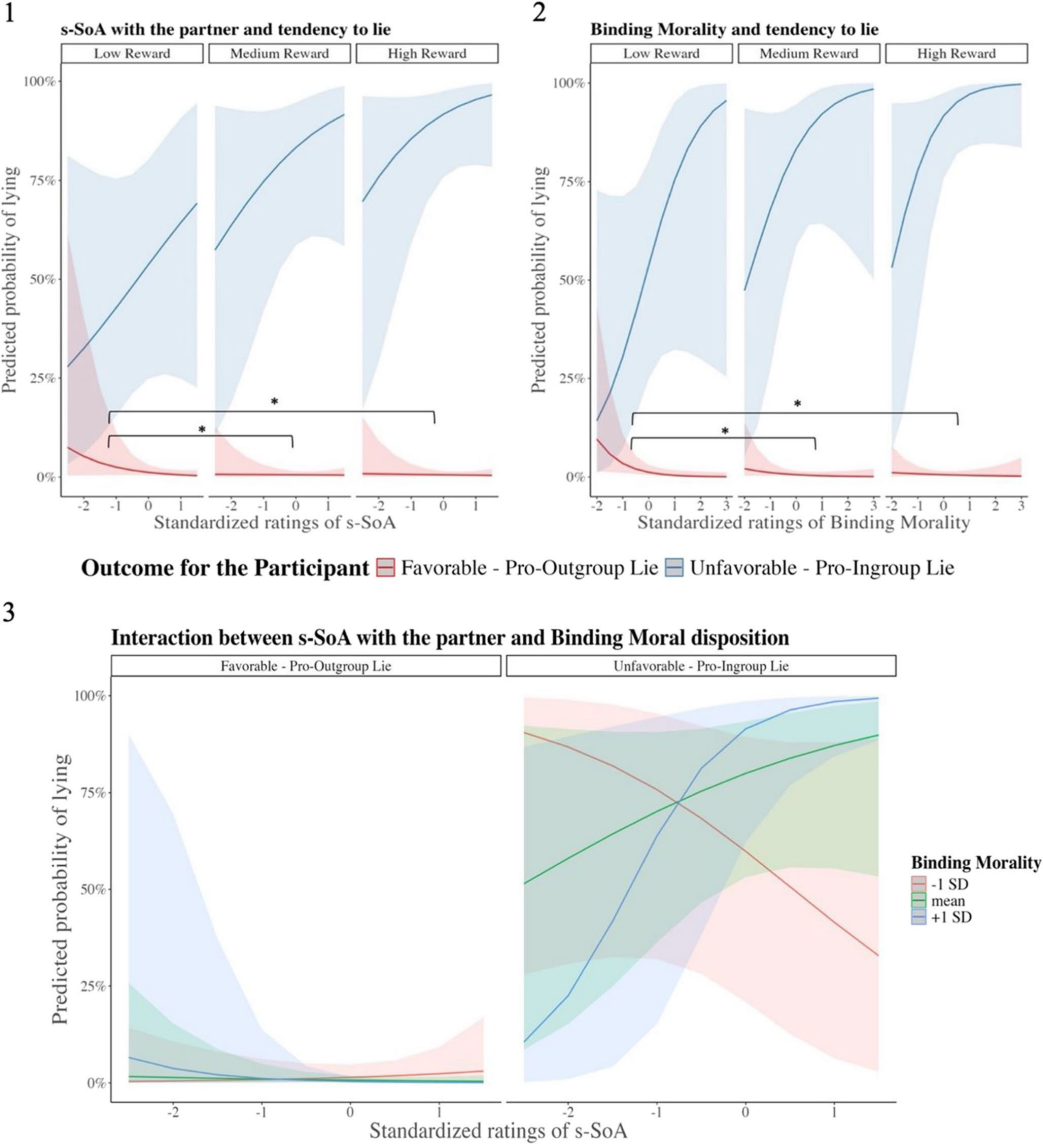
Top images represent the likelihood of lying predicted by the triple interaction between s-SoA, Reward and Outcome (1) and by the triple interaction between Binding Morality (BM), Reward and Outcome (2). In the favorable condition and when rewards were low, participants were more likely to tell pro-outgroup lies for low s-SoA and BM levels, compared to medium and high rewards. Bottom image (3) represents the likelihood of lying predicted by the triple interaction between s-SoA, Binding Morality (BM) and Outcome. In the unfavorable condition, when the opponent team had originally extracted the winning card, the increase of s-SoA was significantly associated with a higher likelihood of lying, but only in participants with high Binding Morality ratings (+1 SD)

## Discussion

This study aimed to explore the influence of shared Sense of Agency (s-SoA) with team members on the tendency to engage in deceptive behaviors in an intergroup context. In the preliminary study, we developed the Tone Tapping Task (TTT), a paradigm designed to modulate s-SoA based on the predictability of a partner. In the main study, we investigated how both the objective predictability of the partner and the subjective experience of s-SoA were associated with intergroup dishonesty. Participants had the opportunity to deceive either to increase their group’s gain or to benefit the outgroup, with each trial associated with varying reward levels (low, medium, or high). We also included a subjective measure of Inclusion of the Other in the Self (IOS) scale, along with participants’ feelings of shared responsibility with their partner during the moral game. These measures allowed us to determine whether s-SoA was more closely associated with a sense of responsibility toward the group or with increased identification with the ingroup partner. Three possible mechanisms were initially proposed to explain the relationship between s-SoA and intergroup dishonesty. The first hypothesis suggested that higher levels of s-SoA would lead to a greater sense of shared responsibility with the partner, thereby reducing intergroup dishonesty. This was based on prior findings indicating that a heightened sense of responsibility for group decisions can discourage harmful behavior toward outgroup members, particularly when individuals are concerned about their group’s moral image (Brambilla et al., 2013) or experience group-based guilt (Halevy et al., 2015). The second hypothesis proposed that higher levels of s-SoA would foster stronger identity fusion with the partner, leading to increased pro-ingroup immoral behavior. This was based on previous research that has shown how engaging in joint tasks enhances social closeness (Tarr et al., 2018), which, in turn, reinforces ingroup identification and promotes parochialism (Balliet et al., 2014; Swann & Buhrmester, 2015). Our third hypothesis suggested that moral dispositions would modulate the relationship between s-SoA and intergroup dishonesty, leading individuals to align with one of the previous two mechanisms based on their moral orientation. Specifically, at higher levels of s-SoA, individuals with lower Binding Morality (BM) scores were expected to exhibit greater ingroup prosociality, consistent with the first mechanism, while those with higher BM scores were expected to display stronger ingroup favoritism, aligning with the second mechanism.

Results of the preliminary study validated the online version of the tone-tapping task (TTT), originally developed by Bolt and Loehr (Bolt et al., 2016; Bolt & Loehr, 2017) as a tool to elicit either strong or weak s-SoA with the interacting partner. We replicated previous findings, with participants reporting higher s-SoA levels after interacting with the predictable partner. In line with evidence suggesting a close link between interpersonal coordination and social closeness (Marsh et al., 2009; Tarr et al., 2018), the predictable partner was also associated with stronger social closeness and identity fusion, measured with the inclusion of the other in the self-scale (IOS). In the main study, we further strengthened the size of the effect by introducing the alternation between the predictable and unpredictable partner, which enhanced the difference between the s-SoA in the blocks that immediately preceded the MP-TLCG sessions (from d = 0.52 to d = 0.89). At variance with what observed in preliminary study, we also found lower p-SoA levels when interacting with the unpredictable partner. Nevertheless, it should be noted that the effect was large (d = 0.89) for s-SoA, while small (d = 0.24) for p-SoA. We speculate that the discrepancy between the findings of the two studies could be explained by a combination of the small size of the effect of partner predictability on p-SoA, and by methodological differences. Indeed, the second study involved a greater sample with respect to the first one, which had an appropriate number of participants to detect the effect of interest on s-SoA but not the small and unexpected effect on p-SoA. Nonetheless, the more prominent impact on s-SoA vs. p-SoA supports the importance of treating them as related but separate constructs and of measuring them separately, rather as two extremes of a continuous measure. Regarding participants’ deceptive behavior, as with other studies that used the TLCG (Panasiti et al., 2011; Parisi et al., 2021), we found that the likelihood of lying increased in the unfavorable outcome condition, thus when the deception increased own group gain. Additionally, in line with previous findings, the size of the possible gain influenced participant behavior (Leib et al., 2021; Scattolin et al., 2022). Indeed, we found a significant interaction between Outcome and Reward, with more pro-ingroup lies and less pro-outgroup lies when the potential gain was higher. Contrary to our hypotheses, we did not observe significant, main or interaction effects connected to the objective differences in partner’s predictability. However, when we included subjective ratings of s-SoA in our analysis, we found a more negative association between both s-SoA ratings and Binding Morality scores with the likelihood of telling a pro-outgroup lie, in trials where participants were exposed to the possibility of obtaining low vs. medium and high rewards. In other words, when either s-SoA or Binding disposition were particularly low, and the possible reward was small, participants were more likely to act against the group’s interest. Interestingly, these modulations were absent when reward levels were medium or high, while pro-ingroup lies remained unaffected. It is possible that higher s-SoA or Binding Morality favored stronger participants’ compliance to the group interest, thus discouraging any pro-outgroup action with detrimental consequences for their own team. An alternative possibility is that participants used pro-outgroup lies as “punishment” towards a partner who they experienced as being unable to create a strong s-SoA. In both cases, the effects of s-SoA and Binding Morality were specific for the lower reward level and were not found in the higher ones, highlighting the crucial role of the magnitude of the monetary incentive. Importantly, we found an interaction effect between s-SoA and Binding moral dispositions over pro-ingroup dishonesty. Post-hoc tests revealed that only in individuals reporting high (+ 1SD) Binding Morality scores, a stronger s-SoA came with an increased likelihood to tell pro-ingroup lies. It should be considered that the interaction was mainly driven by participants having high level of Binding Morality score, with the negative association between s-SoA and pro-ingroup deception in individual having low level of Binding Morality (−1SD) failing to reach significance. Supplementary analysis (S3) suggests that in our study, s-SoA was more strongly associated with IOS than Shared responsibility. Moreover, IOS ratings displayed a pattern of association with moral behavior specular to the one of s-SoA ratings (S4) validating the hypothesis that the effects of s-SoA are linked to stronger identity fusion with the partner. This is in line with previous studies involving synchrony (Tarr et al., 2018), which showed that performing the task with a predictable partner was associated with stronger feelings of social closeness. Moreover, it could then influence the moral behavior, as people tend to favour the close other (Greenwald & Pettigrew, 2014) and to consider morally acceptable favouring close others compared to distant ones (Law et al., 2022). In light of our findings, this can be particularly true for people that strongly emphasize group bonds, as reflected by high levels of Binding Morality (Nilsson et al., 2020).

In summary, our results seem more coherent with the hypothesis that the relationship between s-SoA and intergroup behavior depends on one’s own moral disposition. Indeed, we found no evidence that higher s-SoA level led to lower intergroup dishonesty, as hypothesized in light of the first proposed mechanism. It should be considered that this may also be due to the lack of explicit group reputation concerns in the task, as participants were not informed that outgroup members would learn about their decisions. Without external social pressure to uphold a moral image, the process of shared responsibility may not have been sufficient to reduce intergroup dishonesty in this context. The second proposed mechanism, a general trend of more parochial behavior following an experience of high s-SoA with one’s own group member, aligned only partially with our findings. Participants who experienced lower s-SoA were more likely to lie in favor of the outgroup when the reward was low, compared to when it was medium or high. Yet, no direct effect of higher s-SoA over pro-ingroup deceptions was observed. The third hypothesis, which emphasized the modulatory role of moral disposition, appears to be the most strongly supported by our findings. Specifically, we observed a significant interaction between s-SoA and moral disposition in shaping dishonest behavior. Participants with high Binding Morality (BM) scores were more likely to prioritize ingroup interests, engaging in strategic deception that benefited their own group. Supplementary analysis showed that the dimension of BM more strongly associated with the effect were Purity and Loyalty (Supplementary– S5). The less prominent role of the Authority foundation may be attributed to the task’s structure, which emphasized egalitarian collaboration between coagent and lacked explicit presence of hierarchical roles.

Taken together, our results support the proposed interplay between shared Sense of Agency, moral dispositions, and intergroup dishonesty. They suggest that heightened s-SoA experienced with an ingroup member might amplify parochial deception through increased identity fusion, rather than promoting a stronger sense of shared responsibility—depending on an individual’s moral orientation. Previous evidence demonstrated that individuals may act immorally when such behavior benefits their own group (Cadsby et al., 2016), underscoring how parochial interests can override impartial moral norms. Theoretical frameworks have been proposed to explain such patterns in group-based moral decision-making. For instance, the common moral currency framework (Weisel & Shalvi, 2022) suggests that in situations where competing moral motives are present—such as when lying benefits one’s group but harms another—deception can be construed as the most ethical course of action from the perspective of ingroup loyalty. Our findings expand on this conceptualization by integrating it with Moral Foundations Theory (MFT) and agency research. Since its early formulation, MFT has emphasized the role of binding moral foundations—loyalty, authority, and purity—in sustaining group cohesion and prioritizing ingroup welfare (Graham et al., 2013). This theory also accounts for individual variability in moral priorities, for example in relation to political orientation (Graham et al., 2009) as well as cultural differences (Atari et al., 2022). Concerning intergroup dishonesty, components of Binding Morality—particularly loyalty—play a crucial role, both in isolation and in interaction with contextual factors (Hildreth et al., 2016; Hildreth & Anderson, 2018). Our study contributes to this body of work by demonstrating how individual differences in binding morality interact with situational factors—in particular, the subjective experience of a shared sense of agency with an ingroup member—to influence intergroup dishonesty. This dual-process perspective coherently aligns with prior findings on the interplay between disposition and context in shaping dishonest behavior (Markowitz & Levine, 2021; Panasiti et al., 2011) and provides a more comprehensive understanding of how moral presences are translated in group decision-making settings.

## Conclusion

### Strengths and take-home message

Our findings support the hypothesis that the impact of shared Sense of Agency (s-SoA) on intergroup dishonesty is modulated by moral disposition. This suggests that individuals’ moral frameworks shape how s-SoA influences dishonest behavior in intergroup contexts. Additionally, the study provides insights into the psychological mechanisms underlying ingroup-favoring dishonesty, highlighting the potential role of identity fusion when interacting with a predictable in-group member. Our paradigm also reflects real-world scenarios where dishonest behavior benefits not just the individual but also other members of its group, making the study ecologically relevant. In summary, by highlighting how subjective experiences of agency can activate or dampen the behavioral expression of moral dispositions, our findings offer novel insights into the mechanisms underpinning intergroup morality.

### Limitations

Despite these contributions, our results remain correlational in nature, as the observed effects emerged when analyzing subjective s-SoA ratings rather than the objective manipulation of partner predictability. This indicates that the effects are, at least partially, driven by individual differences in the tendency to experience s-SoA, rather than the actual predictability of a partner’s actions. As a result, our conclusions regarding the impact of objective predictability on intergroup dishonesty are somewhat limited. Future studies should examine whether this lack of effect is a consistent pattern or due to methodological constraints. Another limitation concerns the design of the multiplayer temptation-to-lie card game (MP-TLCG). In our paradigm, the reward was always shared between the participant and their team member, mirroring real-world group deception, where benefits are distributed among group members. However, this structure makes it difficult to differentiate between self-serving dishonesty and group-favoring dishonesty. Future studies should introduce conditions where the partner benefits exclusively from the participant’s deception, as previously explored by Cadsby et al. (2016), to disentangle these motivations.

Additionally, the MP-TLCG structure placed the participant in a decision-making role, with the partner passively involved. This methodological choice ensured that partner behavior did not directly influence participants’ moral decisions. However, it may have reinforced a hierarchical structure, limiting the experience of shared responsibility and emphasizing identity fusion instead. Future studies should explore more interactive group settings where decision-making is distributed among multiple members.

### Future directions and practical implications

Our findings have practical implications for fostering cooperation and ethical decision-making in social and organizational contexts. Encouraging higher levels of s-SoA could be beneficial in strengthening social bonds within teams, potentially improving coordination and cooperation in workplaces or collaborative environments. However, our results also indicate a potential unintended consequence: heightened s-SoA may increase ingroup favoritism, leading to dishonest behavior against outgroups. Raising awareness of this mechanism could help mitigate risks of intergroup discrimination and unethical decision-making. Moreover, our findings emphasize the role of individual moral dispositions in shaping how s-SoA affects intergroup morality. Future research should investigate how moral education, or interventions could be tailored to balance the positive effects of agency-driven group cohesion while preventing its potential misuse for unethical purposes. By understanding the interplay between s-SoA, identity fusion, and moral disposition, interventions can be designed to promote fair and ethical intergroup interactions.

## Electronic supplementary material

Below is the link to the electronic supplementary material.

Supplementary Material 1

## Data Availability

Data are available at the following link: https://osf.io/y7mdx/
