# Supplementary material for: The impact of shared sense of agency and moral dispositions on intergroup dishonesty
Source: Curr Psychol. 2025 Oct 3;44(24):18638–54. doi: 10.1007/s12144-025-08026-0 (PMC12664842; doi:10.1007/s12144-025-08026-0)
Supplement: Supplementary file 1 — Supplementary Material 1 [file 12144_2025_8026_MOESM1_ESM.docx]

Supplementary Information

S1 – Effect of partner predictability in the preliminary study


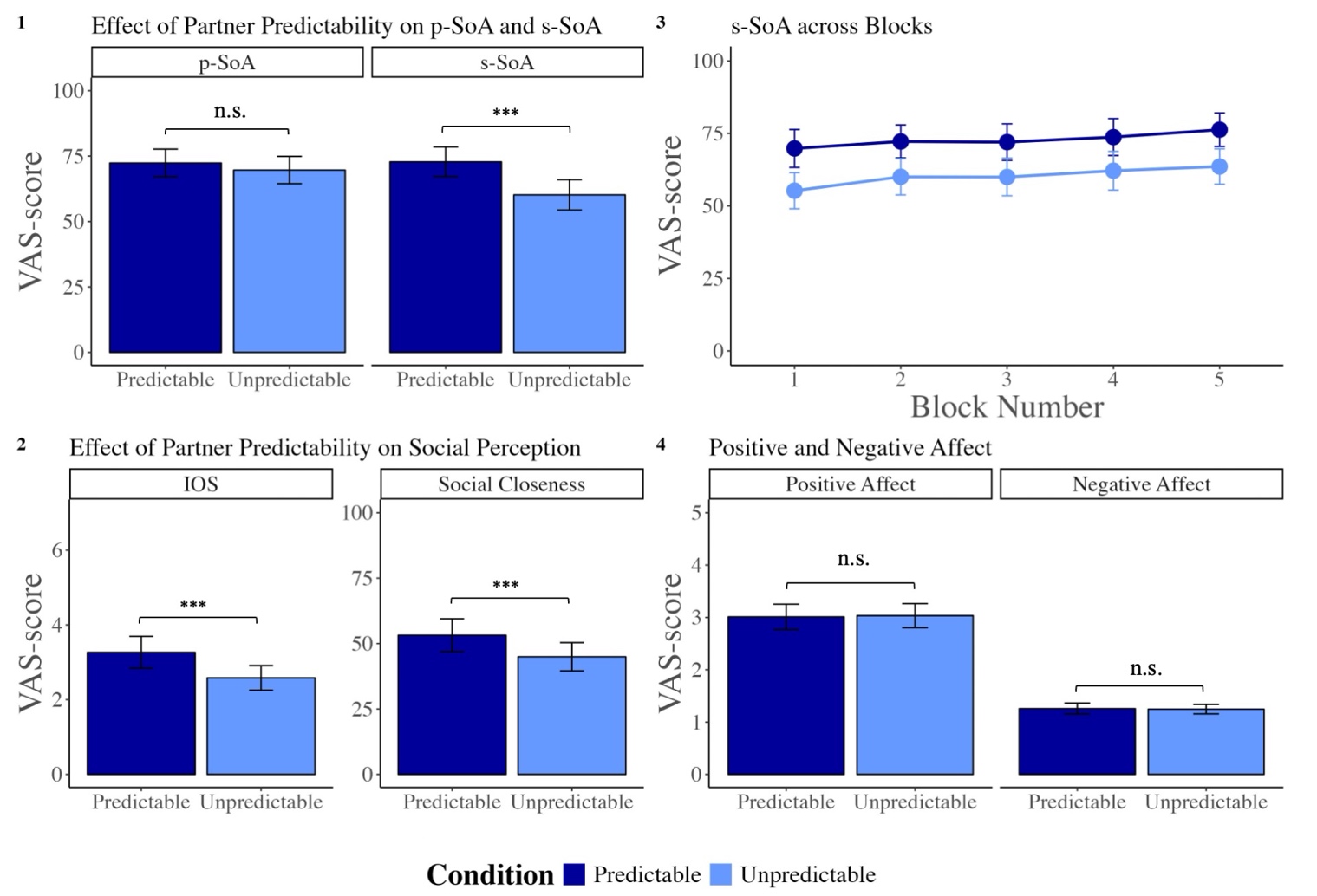


**Figure S1.** Effects of partner predictability on Sense of Agency (SoA), social perception, and affective responses. Dark blue bars and lines represent the predictable partner condition, and light blue bars and lines represent the unpredictable partner condition. Error bars indicate standard errors. (1) The effect of partner predictability on personal SoA (p-SoA) and shared SoA (s-SoA). No significant difference was observed in p-SoA between predictable and unpredictable partners, while s-SoA was significantly higher in the predictable condition compared to the unpredictable condition. (2) The effect of partner predictability on social perception, measured through the Inclusion of Other in the Self (IOS) scale and social closeness ratings. Participants reported significantly higher IOS scores and greater social closeness with predictable partners compared to unpredictable partners. (3) Change in s-SoA across blocks. s-SoA remained relatively stable over time, showing a consistently higher level for predictable partners compared to unpredictable partners throughout all five blocks. (4) The effect of partner predictability on positive and negative affect. No significant differences were found between conditions for positive or negative affect ratings.

S2 – Effects of partner predictability in the main study

S2 – Effect of partner predictability in the main study


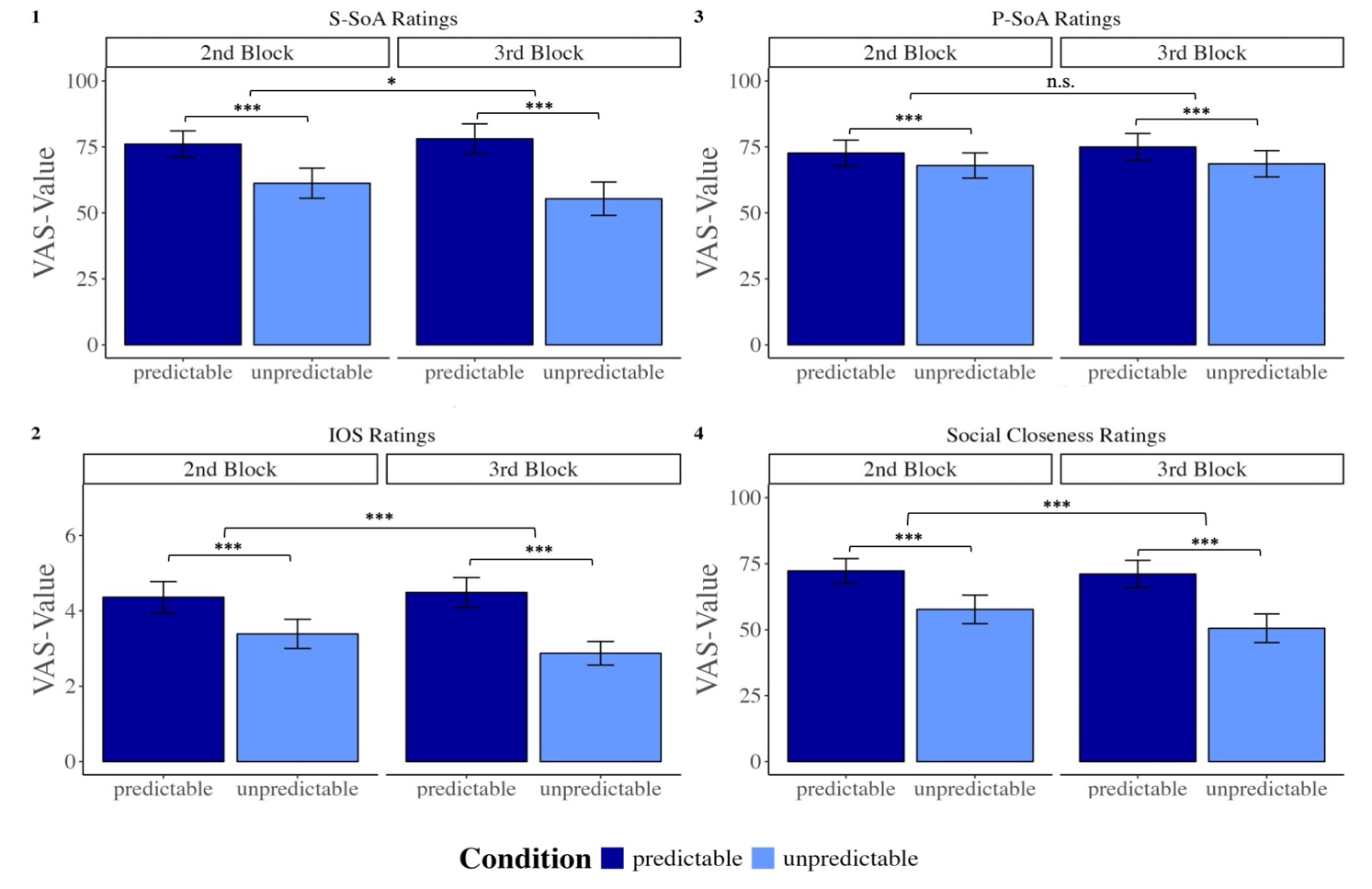


**Figure S2.** Effects of partner predictability on Sense of Agency (SoA), social perception, and social closeness, after the 2nd and 3rd blocks, the latter occurred after having experienced both type of Conditions. Dark blue bars represent the predictable partner condition, and light blue bars represent the unpredictable partner condition. Error bars indicate standard errors. (1) Shared Sense of Agency (s-SoA) ratings in the 2nd and 3rd blocks. Participants reported significantly higher s-SoA when interacting with a predictable partner compared to an unpredictable partner, with a larger difference after the third block (2) Inclusion of the Other in the Self (IOS) ratings in the 2nd and 3rd blocks. Participants perceived greater inclusion of the partner (IOS) in the predictable condition compared to the unpredictable condition, with a larger difference after the third block (3) Personal Sense of Agency (p-SoA) ratings in the 2nd and 3rd blocks. Participants reported significantly higher p-SoA in the predictable condition compared to the unpredictable condition, similarly across blocks. (4) Social closeness ratings in the 2nd and 3rd blocks. Participants reported significantly greater social closeness with a predictable partner compared to an unpredictable partner, with a larger difference after the third block.

S3 – Association of s-SoA with IOS and Shared Responsibility

We run supplementary analysis to check whether increase in s-SoA was associated with IOS and Shared Responsibility during the moral task. We found that IOS post MP-TLCG was stronger towards the predictable partner compared to unpredictable partner (b = 0.7, t = 4.85, p < 0.001), while Shared Responsibility displayed no significant modulation (p = 0.21). We then checked whether the mean scores of s-SoA over the two conditions were associated with the mean score of IOS and Shared responsibility. IOS displayed a significant correlation (r = 0.32, p = 0.006), while the correlation with Shared responsibility did not reach significance (p = 0.067).


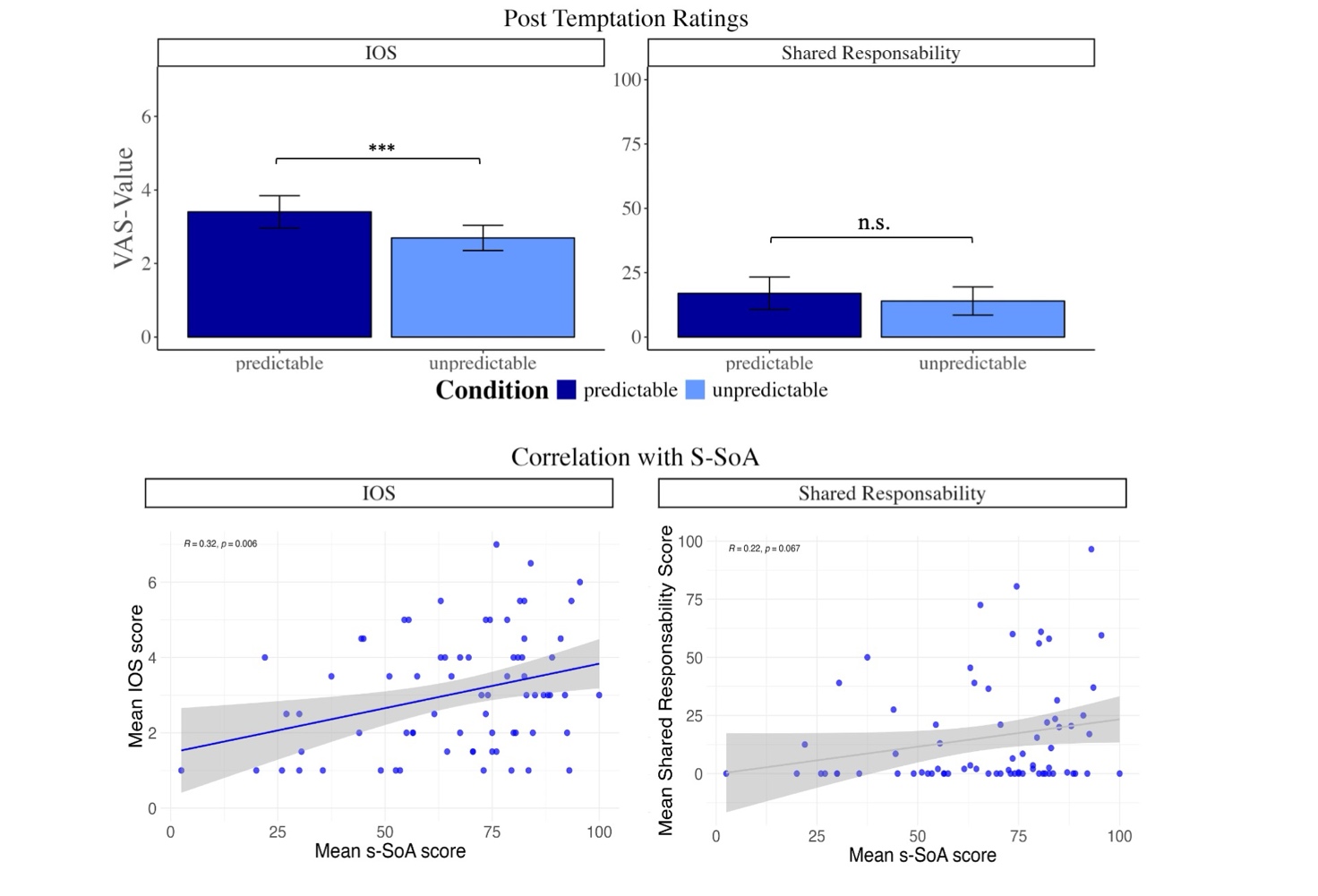


**Figure S3.** Effects of partner predictability on IOS and Shared Responsibility ratings after the Multiplayer Temptation to Lie Card game. The top panels display the ratings for both conditions. Similar to s-SoA, IOS scores were higher in the predictable condition. In contrast, Shared Responsibility did not show any significant differences between conditions. The bottom panels display the correlation between the mean s-SoA score and the mean IOS and Shared Responsibility scores. Only the IOS score showed a significant correlation with s-SoA, while the correlation with Shard Responsibility did not reach significance.

S4 – IOS and Moral behavior


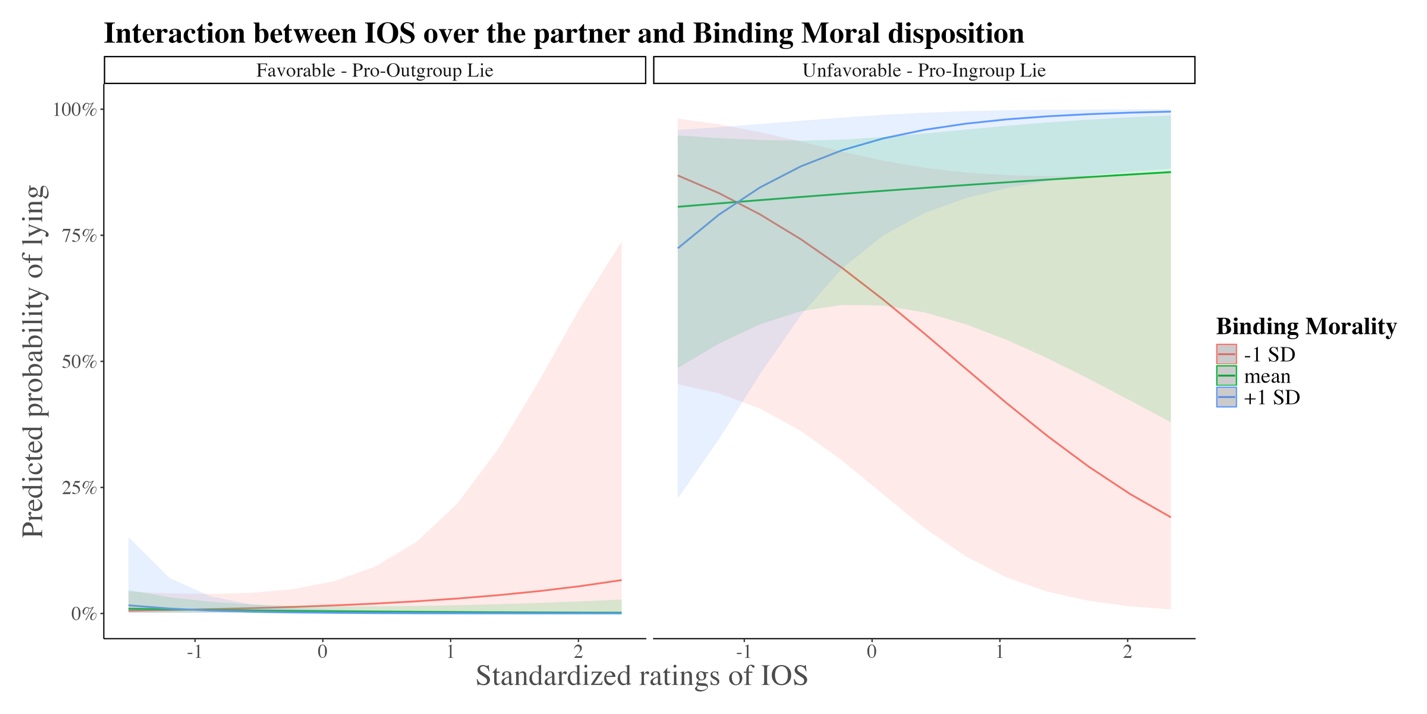
We found significant triple interactions between IOS, Outcome and Binding Morality (BM) (χ2 = 8.27, p = 0.004) (See Figure S4). The post-hoc comparisons revealed that the increase in dispositional ingroup Binding morality was associated with the increase of the positive relationship between IOS and the likelihood to lie in the unfavorable condition (z = 2.54 p = 0.011). Such positive relationship was significant only for people with high dispositional BM (+1 SD), for whom the increase of IOS came with a higher likelihood of telling pro-ingroup lies (estimate = 1.13, SE = 0.57, z = 2.26, p = 0.047). We also found that the increase of dispositional ingroup Binding morality was associated with an increase of the negative relationship between IOS and the likelihood to lie in the favorable condition (z = 2.16, p = 0.031). Such negative relationship was significant only for people with high dispositional BM (+1 SD), for whom the increase of IOS came with a lower likelihood of telling pro-outgroup lies (estimate = -1.16, SE = 0.80, z = -1.99, p = 0.046).

**Figure S4**. Relationship between IOS and moral behavior. The plot represents the likelihood of lying predicted by the triple interaction between IOS, Binding Morality (BM) and Outcome. Similarly to what found using s-SoA ratings, in the unfavorable condition, when the opponent team had originally extracted the winning card, the association between IOS and the likelihood of lying, was modulated by Binding Morality ratings. In the unfavorable condition, when the opponent team had originally extracted the winning card, the increase of IOS was significantly associated with a higher likelihood of lying, but only in participants with high Binding Morality ratings (+1 SD). In the favorable condition, when the opponent team had originally extracted the losing card, the increase of IOS was significantly associated with a lower likelihood of lying, but only in participants with high Binding Morality ratings (+1 SD).

S5 – Subdimensions of Binding Morality and Interaction with s-SoA


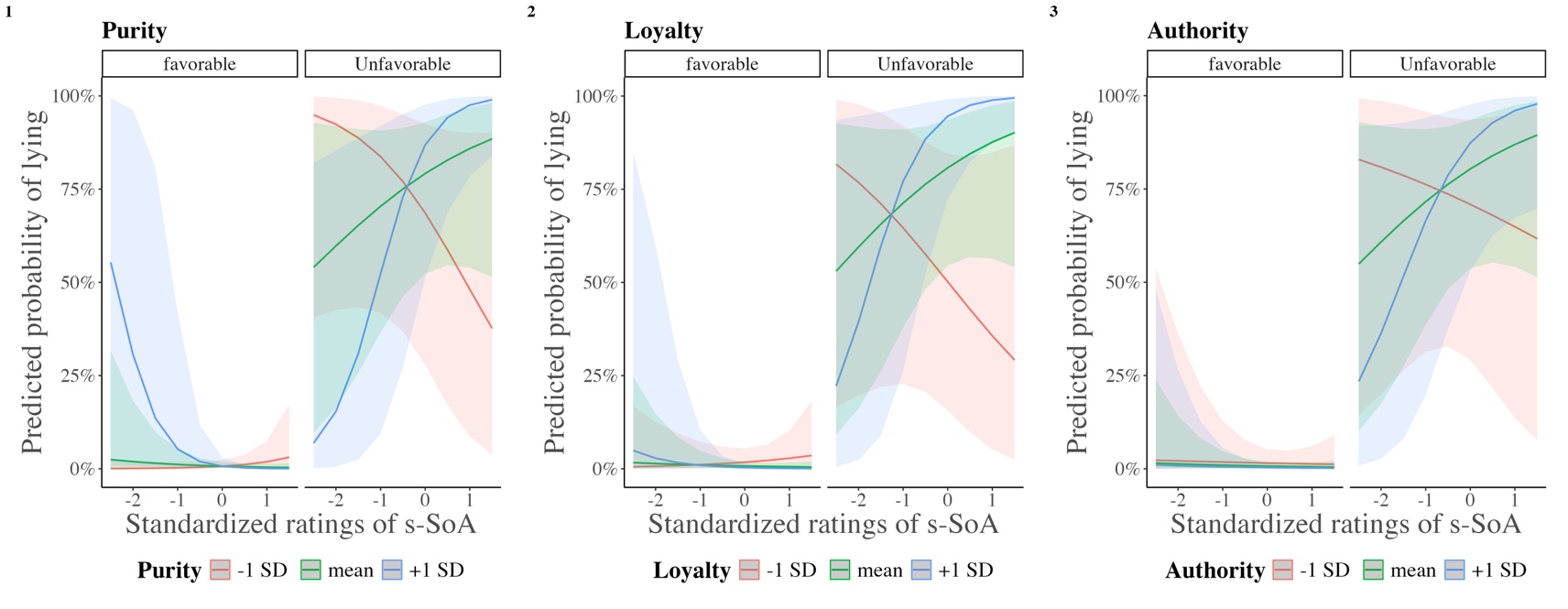
We independently examined the role of the three subdimensions of Binding Morality—Loyalty, Purity, and Authority—in modulating the association between shared Sense of Agency (s-SoA) and dishonest behavior. A significant three-way interaction between s-SoA, Outcome, and the moral foundation emerged for Purity *(*χ2 = 7.56, p = 0.005*)*, a marginally significant effect for Loyalty *(*χ2 = 3.76, p = 0.052*)*, and no significant interaction for Authority *(*χ2 = 0.84, p = 0.36*)*. Post-hoc comparisons showed that increases in both Purity *(z = 2.53, p = 0.011)* and Loyalty *(z = 2.29, p = 0.026)* were associated with a stronger positive relationship between s-SoA and the likelihood of lying in the Unfavorable condition. For both Purity and Loyality such positive relationship was significant only for people with high dispositional scores (+1 SD), for whom the increase of s-SoA came with a higher likelihood of telling pro-ingroup lies (Purity: estimate = 1.80, SE = 0.76, z = 2.36, p = 0.018; Loyalty: estimate = 1.65, SE = 0.76, z = 2.17, p = 0.030). Notably, only Purity also modulated the relationship between s-SoA and the likelihood of lying in the Favorable condition (*z = 2.77, p = 0.006*). This association was significant exclusively for individuals with high Purity scores (+1SD), for whom the increase of s-SoA came with a lower likelihood of telling pro-outgroup lies (estimate = -2.07, SE = 0.86, z = -2.40, p = 0.016; In contrast, no significant effects were observed for Authority in any condition (all p > 0.07).

**Figure S5**. Relationship between subcomponents of Binding Morality and moral behavior. The plot represents the likelihood of lying predicted by the triple interaction between Outcome, s-SoA and 1) Purity, 2) Loyalty and 3) Authority. Similarly to the findings obtained by using the composite Binding ratings, in the unfavorable condition, when the opponent team had originally extracted the winning card, the association between s-SoA and the likelihood of lying, was modulated by Purity and Loyalty ratings, while Authority rating did not display significant associations. In the unfavorable condition, when the opponent team had originally extracted the winning card, the increase of s-SoA was significantly associated with a higher likelihood of lying, but only in participants with high Purity or Loyalty ratings (+1 SD). In the favorable condition, when the opponent team had originally extracted the losing card, the increase of s-SoA was significantly associated with a lower likelihood of lying, but only in participants with high Purity ratings (+1 SD).
